# Preventing surgical disputes through early detection and intervention: a case control study in China

**DOI:** 10.1186/s12913-014-0671-5

**Published:** 2015-01-22

**Authors:** Ping Xu, Zhenlin Fan, Ting Li, Lijie Wang, Qingwen Sun, Xia Du, Bin Lian, Lulu Zhang

**Affiliations:** Department of Health Service Management, Second Military Medical University, 800 Xiangyin Road, 200433 Shanghai, China; Department of Medical Education, Eastern Hepatobiliary Surgery Hospital, Shanghai, China; Department of Mathematics, Second Military Medical University, Shanghai, China; Eastern Hepatobiliary Surgery Hospital, 225 Changhai Road, 200433 Shanghai, China

**Keywords:** Medical disputes, Prevention, Early detection, Intervention, Surgical treatment

## Abstract

**Background:**

Medical disputes have become a serious issue in China. A crisis cannot usually be predicted and managed through a cost–benefit strategy; therefore, researchers believe that prevention is better than containment and post-crisis resolution. This study aimed to identify solutions to prevent medical disputes in surgical cases through early warning and intervention of potential cases.

**Methods:**

A case–control study was conducted to identify early detection indicators of medical disputes in the surgical treatment of liver cancer through Delphi consultation and logistic regression on the basis of which interventions were undertaken to prevent potential cases.

**Results:**

The dispute detection model was composed of patient age (P = 0.08), frequency of hospitalization (P = 0.003), length of hospital stay (P < 0.001), terminal condition (P = 0.004), unplanned reoperation (P = 0.048), blood transfusion volume (P = 0.006), and arrearage (P < 0.001). Risk management interventions through quality improvement and enhanced communication in cases with an abnormal performance indicator proved effective in practice.

**Conclusions:**

This study explored the use of an evidence-based medical risk management strategy for medical disputes that involved early detection and intervention and could potentially be adopted by hospitals to prevent medical disputes.

**Electronic supplementary material:**

The online version of this article (doi:10.1186/s12913-014-0671-5) contains supplementary material, which is available to authorized users.

## Background

There is a growing trend towards increased complaints and claims against healthcare providers that result in medical disputes. This issue has evolved into a special global issue in healthcare. Studies have shown that medical disputes arise mainly from medical errors/malpractice, miscommunication, and an over-emphasis on cost containment that leads to fewer treatment resources within the health system [1,2]. However, some researchers believe that mistrust between patients and providers is another contributor to the rising number of medical disputes [3]. The patient’s lack of adequate information, combined with the provider’s reluctance to provide prompt and clear explanations, can lead to conflict, especially when there is an unfavorable outcome.

Medical disputes not only undermine the relationship between patients and providers, but are also extremely costly to the healthcare system, thus adversely impact the quality of care by limiting available resources. Professional liability insurance for physicians and hospitals that is allocated to cover litigation or torts costs between US$76 to $122 billion per year [4]. In a Japanese study, it was reported that 87.1% of dispute cases were awarded compensation with an average amount of US$38,937, and 38.1% of cases judged not to involve error were also compensated a total of US$1,000,000 to resolve disputes [5]. In addition to the expenditures on litigation and compensation, maintaining a risk management system within health facilities dedicated to handling possible crises also increases administrative costs [6].

The situation has become more serious in China because of deficiencies in the healthcare system and the poor medical literacy of the general public. In recent years, medical dispute has grown into one of the most serious health issues in China. This issue has led to growing tensions within the doctor–patient relationship and has also prompted violence against doctors. Because of the negative social impact and adverse consequences [7,8], immediate intervention is required [9,10].

While litigation, arbitration and third-party mediation are the main avenues available to resolve medical disputes [2], these methods often have disadvantages from the point of view of economics, efficiency, and in some cases legal perspectives. Consequently, monetary compensation is usually the only solution to resolving the dispute [11].

As crises usually cannot be predicted and managed through a cost–benefit strategy, researchers believe that prevention is better than post-crisis containment [12]. As a result, a well-designed, strategy- and institutional-based risk management program has been recommended as a means of preventing crises to decrease the number of medical disputes [6,13].

However, practical studies on the issue are limited, especially in China; therefore, the implementation of hospital risk management strategies for the resolution of medical disputes is encouraged. This study aimed to establish a risk management system based on early detection and intervention of potential dispute cases to prevent them from occurring and to improve the quality of medical care.

According to official statistics, during 2008–2010, the majority (39.63%) of medical disputes in China occurred in surgical departments in Shanghai, China. This finding probably stems from the large number of invasive and high-risk surgical procedures performed in Shanghai that are associated with severe complications and unfavorable outcomes.

As a disease category could be the basic unit of quality assessment in a large hospital [14], the current preliminary study focused on dispute cases that involved surgical treatment of liver cancer. Dispute cases involving liver cancer were chosen because they represent cases with the highest incidence, longest duration, poorest prognosis of the patient, and highest treatment costs in China [15].

## Methods

The study was a retrospective, case–control design to support evidence-based management practices. Key indicators of medical disputes were first determined through a literature review, expert consultation, and statistical analyses based on patient medical records. The early detection system for potential disputes was then established with the above indicators, and related interventions were applied to prevent the crisis.

### Ethics statement

The study was approved by the Ethics Review Board of Second Military Medical University, Shanghai, China, with the reference number of 2008LL023.

### Retrospective review of medical records

In the preliminary study, data were collected from the largest hepatobiliary surgery center in Shanghai, China. Fifty-three severe surgical dispute cases related to the treatment of liver cancer reported from January 2004 to December 2008 were collected as the case group (Table 1), and 145 comparable non-dispute cases during the same period and with similar diseases were included in the control group. All of the information for these cases was obtained through a retrospective review of patient medical records.

**Table 1:** **Distribution of cases included in the study**

**Years**	**Surgical cases**	**Dispute cases**	**Percentage (%)**
2004	1894	3	0.16
2005	1984	7	0.35
2006	2420	10	0.41
2007	2791	17	0.61
2008	2928	16	0.55
Average	2403	10.6	0.42

### Identifying indicators of medical disputes

The study summarized possible risk factors for medical disputes in surgical treatment that were applicable to the situation in China by reviewing the literature using the following types of key words: “medical disputes”, “medical risk”, “indicator”, “factors”, and “causes” [16–29]. The primary indicator system included more than 30 alternatives sorted by categories that were related to the patient, provider, disease, communication, management, and hospital and societal environment.

A two-round Delphi consultation was held to differentiate the key indicators from the primary ones. Fifteen experts with backgrounds in clinical care, management, statistics, epidemiology, health economics, social health, and psychology participated using the Like 5 importance scaling method. During the process, expert suggestions were also obtained and taken into consideration to modify the system. The scale and variation coefficient of the selected indicators was determined to be above 3.5 and below 0.25. A final agreement was reached for 19 potential indicators. For detailed information about the Delphi process and results please refer to Additional file 1.

### Data analysis

Information about indicators obtained from medical records for both the case and control groups was used to assemble a database in Excel 2007 (Microsoft, Redmond, WA, USA) that was analyzed using SPSS version 18.0 (Statistical Package for the Social Sciences; IBM, Armonk, NY, USA).

The indicators were converted into categorical variables and several thresholds were set for the purpose of analysis as follows: as recommended by the World Health Organization, an age of 45 years was used to distinguish the young from middle-aged and older people. Considering that 90% of inpatient stays without disputes were less than 30 days, the variable threshold for hospital stay was set at 30 days. A terminal condition notice issued to patients by their physician signaled a critical situation. An unplanned reoperation would not occur under normal circumstances; therefore, it was categorized as Yes/No. Surgical bleeding was considered abnormal when bleeding or the transfusion volume exceeded 2,000 ml. Hepatectomies were considered major when the diameter was over 5 cm. Finally, 20,000 RMB was taken as the warning threshold that reflected arrearage, which was unusual in ordinary cases.

A total of 198 cases were entered into the database and were checked twice. The analysis included 53 disputes (26.77%) and 145 non-dispute cases (73.23%) for each indicator. Multivariate logistic regression was used to identify significant variables with 0.1 and 0.15 as the inclusion and exclusion criteria, respectively. A regression model of key indicators for medical disputes was then established.

### Empirical study

The empirical study was carried out from January 2009 to December 2012. Significant variables derived from the regression model were applied with daily monitoring to prevent medical disputes related to surgical treatments. The indicator system was merged into the hospital management information system to obtain real-time data collection and feedback. Early interventions were instituted in cases with an abnormal indicator performance.

## Results

### Indicators with significance for medical disputes

A greater number of men (83.33%) than women (16.67%) were in both the dispute and non-dispute groups (P = 0.026). The two groups did not significantly differ in age, sex, marital status, employment, and residency (P > 0.05).

The regression model of significant indicators was established to predict possible medical disputes through a multivariate analysis. Ultimately, seven indicators: frequency of hospitalization, age, length of hospital stay, terminal condition, unplanned reoperation, blood transfusion volume, and arrearage were included in the model. Among the variables, frequency of hospitalization refers to inpatient times of patients for the cancer, as many patients had been admitted to other hospitals before. Length of hospital stay refers to inpatient days; the longer the patient stays the greater the possibility that they encounter complicated situations. A terminal condition refers to a physician issuing a notice to claim the critical condition of the patient. Unplanned reoperation refers to a patient receiving an unplanned reoperation during the same hospitalization as a result of direct or indirect complications of the surgical procedure. Blood transfusion volume during operation reflects bleeding status and predicts prognosis. Public healthcare is not free in China and patients still need to pay an amount of money even if they are covered by public health insurance. A deposit of 35,000 RMB is required by the hospital from patients; the leftover is recovered after discharge. However, when expenditure exceeds this amount before discharge, a larger deposit is sought. In some circumstance, such as dissatisfaction or mistrust, patients or their family members refuse to pay in time and then the arrearage occurs.

The model is:

$$ \begin{array}{l} \log \frac{P\left(Y=1\right)}{1-P\left(Y=1\right)}=-1.617-0.987{X}_{{}_{frequency\  of\  hospital ization}}-0.928{X}_{{}_{age}}+3.610{X}_{{}_{length\  of\  hospital\  stay}}+1.541{X}_{{}_{terminal\  condition}}\\ {}+1.138{X}_{{}_{unplanned\  reoperation}}+1.629{X}_{{}_{blood\  transfusion}}+4.579{X}_{{}_{arrearage}}\end{array} $$

(Table 2).

**Table 2:** **Logistic regression analysis of the variables for medical disputes**

**Variables**	**B**	**Std.E**	**Wald**	**df**	**P**	**Exp(B)**	**95.0% C.I. for EXP(B)**	
Constant	−1.617	0.666	5.889	1	0.015			
Hospitalization frequency	−0.987	0.330	8.913	1	0.003	0.373	0.195	0.713
Age	−0.928	0.530	3.062	1	0.080	0.396	0.14	1.118
Hospital stays	3.610	0.723	24.967	1	0.000	36.982	8.973	152.419
Terminal conditions	1.541	0.535	8.289	1	0.004	4.667	1.635	13.321
Unplanned reoperation	1.138	0.576	3.903	1	0.048	3.120	1.009	9.645
Blood transfusion volume	1.629	0.592	7.577	1	0.006	5.098	1.599	16.260
Arrearage	4.579	1.037	19.510	1	0.000	97.394	12.769	742.855

**Figure 1:**
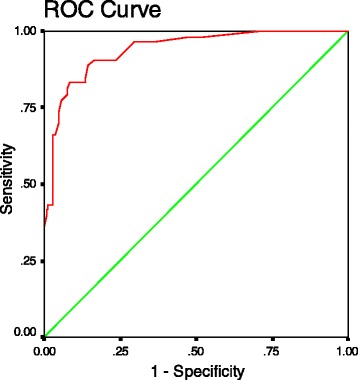
**The receiver operating characteristic curve of the dispute model**.

Frequency of hospitalization and age were negatively correlated with the occurrence of disputes, while arrearage had the largest impact on the model, suggesting that there was a close association between medical expense and doctor–patient conflicts. The model fit well with a sensitivity of 94.5% and a specificity of 77.4% (Table 3). The area under the curve of the receiver operating characteristic curve was 0.938 (95% CI: 0.902–0.974, Figure 1).

**Table 3:** **Simulation results of the model**

**Actual**	**Simulation**			**Accuracy (%)**
	**Non-disputes**	**Disputes**	**Total**	
Non-disputes	137	8	145	94.5
Disputes	12	41	53	77.4
Total	159	49	208	89.9

### Preventing potential medical disputes through early risk management

**Figure 2:**
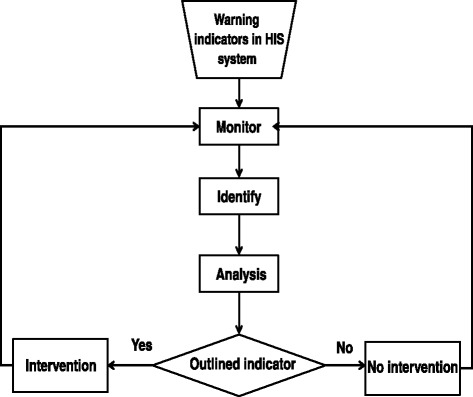
**The process of the early risk management system for potential disputes.**

An empirical study was conducted with a systematic framework for the medical risk management of potential medical disputes that involved dispute indicator monitoring, identification, analysis, and risk intervention (Figure 2).

Based on the early detection indicator model for disputes, a health risk report system was established and was inserted as part of the hospital management information system. This system helped to institute real-time monitoring, detection, judgment, analysis, and evaluation of dispute risk indicators, the results of which were fundamental to management activities.

From January 2009 to December 2012, the intervention strategy included the establishment of a medical risk management committee, the application of targeted measures on key risks to control clinical quality, and the adoption of administrative communication with patients.

#### Medical risk management committee

A three-level management committee was set up specifically for the early detection of medical risks and was composed of a leading committee, an administrative section, and a branch group in each clinical department. Members of the leading committee included the vice dean of the hospital, the head and experts from the departments of administration, and representatives from quality management, nursing, infection control, information, and medical records who were responsible for making decisions. The administrative section for risk detection was part of the hospital’s administration department and was in charge of monitoring, detecting, judging, and evaluating medical risks as well as exploring intervention strategies. Performance indicators were real-time monitored and reported weekly to the entire hospital by each section. Branch groups within each clinical department traced work daily and reported any risk situation to the administrative section.

#### Intervention for key risks to ensure quality

Intervention measures were applied to potential dispute cases with indicators that reached or exceeded thresholds. To avoid malpractice and promote quality treatment, examples of measures that were taken included the following: special attention to patients who refused to pay, with the aim of discovering the reason for the refusal; the arrangement of a consultation with experts for patients with extraordinarily long hospital stays to analyze the problem(s) and improve treatment; investigation of cases in which patients received a blood transfusion volume exceeding 2000 ml, with close monitoring of vital signs and the degree of hemostasis; preoperative discussion prior to an unplanned reoperation in the department to make a comprehensive judgment on the case and outline details according to guidelines, followed by careful monitoring of the patient post-operatively; special attention to the indications for each surgical procedure for patients receiving a terminal condition notification; and psychological support for young and middle-aged patients with a first-time hospital admission.

#### Administrative communication with patients

Facilitating patient–provider communication is another approach to prevent medical disputes. The routine talk before surgery was conducted by the attending. Special conversations were held by the administrative staff with patients or their families in cases with abnormal indicator values to promote understanding concerning reasonable prognostic expectations of the disease and treatment outcome. The two-round administrative conversations were led by the administrative department and involved the chief, attending and resident doctors, as well as patients and key family members. The process was recorded by audio and video. During the conversation, the medical and administrative staff explained the need for and limitations of invasive procedures, the possible outcomes and complications, as well as the legal responsibility of both parties. In addition, requirements and questions from patients or their families were collected to finalize the treatment plan that was agreed upon by both parties.

### Evaluating the early detection indicator system in practice

After applying key indicators for the early detection and intervention of medical disputes among patients who underwent surgical treatment for liver cancer for 4 years, the dispute rate declined significantly irrespective of the complexity of the surgical cases, confirming the success of this approach (Table 4).

**Table 4:** **The effect of the early detection indicator system on dispute rates**

**Intervention**	**Years**	**Surgical cases**	CD rate* (%)	**Dispute rate**	**P**
Without	2004	4133	63.8	0.12	0.014
	2005	4828	64.1	0.17	
	2006	5582	63.9	0.18	
	2007	5992	65.0	0.28	
	2008	6372	65.2	0.25	
With	2009	6710	65.5	0.09	
	2010	6741	69.1	0.07	
	2011	7318	73.0	0.07	
	2012	7442	75.2	0.08	

*CD rate refers to the percentage of surgical cases classified into categories C and D according to the disease severity of patients [14].

## Discussion

The quality of clinical care and effective patient–provider communication are believed to be crucial to avoid malpractice and disputes in hospitals. Unlike the situation in Western countries [30], an increasing rate of disputes in China are mainly a result of deficiencies within the healthcare system. Consequently, paying greater attention to potential dispute cases by monitoring risks factors and improving the quality of treatment, communication, and understanding with patients, would also offer solutions from the provider’s perspective. This approach could prove especially useful as the healthcare system in China is currently in the process of restructuring.

### Early detection of potential disputes with key indicators

It is more cost-effective to prevent a medical dispute than to solve it after it has occurred. The core idea behind this study is that the foundation of effective prevention is the early detection of possible cases so that targeted intervention measures can be taken to avoid a worsening situation.

Through Delphi consultation and statistical verification, a preliminary study established the indicator system for the prediction of medical disputes in surgical cases involving liver cancer, based on historical data. Due to the availability of the data, selected indicators might be intermediate and not necessarily lead to disputes. However, they suggest the potential for disputes to develop because of an unfavorable clinical outcome and are easy to trace in daily work, which is a reflection of their application value in practice.

According to the model, age, frequency of hospitalization, length of hospital stay, terminal condition, unplanned reoperation, blood transfusion volume, and arrearage were key indicators related to medical disputes in surgical cases involving liver cancer. Clinically, a terminal condition, unplanned reoperation, and blood transfusion volume, which are positively related to disputes, usually reflect complicated situations for patients that may lead to unfavorable outcomes and thus possible disputes. Long hospital stays also signaled the possibility of more complications and undermined the impact of rehabilitation after surgery. The negative impact of hospitalization frequency and age on the model was possibly because of better preparation and lower expectations from patients and their families in cases in which the patient’s age and lengthy hospitalizations were significant factors. Arrearage had more administrative than clinical implications. Shortage of money because of inadequate health insurance or economic difficulty, and refusal to pay because of dissatisfaction, were the two main reasons contributing to arrearage. As a result, arrearage could reflect negative attitudes exhibited by patients, which is important to note for early prevention of a crisis.

### Resolving the problem of medical disputes through risk detection and management

The early detection and management system for dispute risks was composed of an indicator analysis and early intervention. Indicator analysis involves monitoring, detecting, analyzing, judging, and evaluating medical risks to provide clues and early warning signals of possible disputes. It is based on this evaluation that prevention strategies can take a crucial role in crisis management.

Sources of conflict can be grouped into four main categories: data mismatch, resource issues, emotional or values-based issues, and communication [31]. From the perspective of providers, improving the quality of healthcare and facilitating mutual understanding with patients represent potential solutions that can avoid malpractice by covering the information gap and promoting trust from patients and their families. Among the interventions undertaken in this study, additional emphasis on potential dispute cases above and beyond ordinary quality control helped to decrease mistakes that resulted from neglect. Furthermore, conversations with administrative and clinical staff had a positive influence on the attitudes of patients and their families toward the outcome.

### Monitoring potential medical disputes using the hospital information system

By combining an indicator system with a hospital management information system, real-time monitoring promoted greater efficiency and effectiveness of hospital management in handling disputes. The system not only helped to uncover target cases for intervention but also provided a pathway with which to reflect on problems and risks in daily clinical practice, thus forming a medical risk prevention mechanism [32].

### Promoting medical quality through evidence-based management

Unlike the current outcome-oriented management mode for medical disputes in China [33,34], this study adopted an evidence-based research design [35,36] by performing a case–control analysis of dispute and non-dispute groups to determine indicators. Although making decisions based on well-designed research is the preferred choice for improving efficiency and effectiveness in health administration, evidence-based methods have not been extensively used in the field of medical management in China. As a pilot, this study suggests an effective and practical resolution to health dispute problems based on scientific exploration and empirical verification.

### Limitations

It is generally agreed that many factors, such as the environment, social, economic and medical literacy of patients, communication, and physician attitudes etc., contribute to the occurrence of disputes. However, indicators in the current model were all disease-related and cases with high risks were all in a state of severe disease. The purpose of this study was to impose early intervention to prevent health disputes in hospital. Therefore, it is still very important to have this quantitative model for targeting patients who are prone to disputes in daily hospital management when other information is unavailable. The study adopted a retrospective approach using data from patient records of only one hospital, which may lead to selective bias and is not representative of the whole population. The design relied on the existing database of patient records in the hospital so that the attainable variables were limited. As a result, we may have missed some important indicators. The use of static data could also lead to a loss of complete information related to dispute cases. Moreover, despite the high reliability (both sensitivity and specificity) of the indicator model, the validity may be questioned. First, owing to limitations imposed by time constraints, manpower, and data availability, we only focused on surgical cases involving liver cancer in a special hospital. Second, differences between patients, disease severity, and treatment process in different hospitals (e.g., general hospitals) could contribute to different dispute indicators. In addition, indicator threshold values were partly based on the experience of experts; therefore, the appropriateness of these threshold values needs to be further investigated. As a result, the indicator system and thresholds need critical testing before they can be widely adopted. Despite the deficiencies mentioned above, the study has explored a more cost-effective way of solving the problem of health disputes in surgical treatment.

## Conclusions

This study investigated early detection indicators that support the prevention of possible medical disputes in the surgical treatment of liver cancer through quality improvement and communication enhancement. Furthermore, it represents an important pilot study in the practice of evidence-based medical risk management in China.

## Additional file

Additional file 1:
**Expert opinions on the indicator system.**
