# Supplementary material for: Preventing surgical disputes through early detection and intervention: a case control study in China
Source: BMC Health Serv Res. 2015 Jan 22;15:5. doi: 10.1186/s12913-014-0671-5 (PMC4312442; doi:10.1186/s12913-014-0671-5)
Supplement: Additional file 1: — Expert opinions on the indicator system. [file 12913_2014_671_MOESM1_ESM.docx]

Additional file 1: expert opinions on the indicator system

The study summarized many factors related to medical risks in hospitals based on a literature review, Delphi consultation and statistical analysis (Table A1).

Table A1. Medical risk factors in hospital before consultation.

| Category | Factors |
| --- | --- |
| Patient | Age, gender, marriage status, occupation, income, residency, insurance, attention to disease, medical knowledge, outcome expectation, awareness of rights |
| Provider | Professional knowledge, skills, professional ethics, responsibility, attitude, legal consciousness, risk awareness, workload |
| Disease | Diagnosis, frequency of hospitalization, hospital stays, admission form, major/minor hepatectomy, preoperative Child-Pugh classification, the presence of additional surgical procedures, therapeutics, complications, bleeding volume in procedure, volume of blood transfusion |
| Communication | Patient-provider communication |
| Management | Executive of clinical regulation and guidelines |
| Hospital environment | Hospital facilities and layout |
| Social environment | Mass media report, public opinion, laws and regulations |

First round consultation:

Nine experts suggested excluding qualitative factors in the provider, communication, management, hospital, and social environment categories, while another six proposed deleting “attention to disease” , “medical knowledge” , “outcome expectation,” and “awareness of rights” from the patient category. The rest of the indicators related to patient and disease situation were grouped into three categories: patient information, hospitalization situation, and discharge situation.

Eight experts suggested including “unplanned reoperation” because it was greatly related to clinical risks.

Four clinical experts considered procedure type, times of emergency treatment, and admission to the intensive care unit (ICU) that were also closely related to high medical risks.

Two nursing experts proposed “care level.”

Six experts suggested clarifying “complications” as “surgical complications” and “non-surgical complications”. Surgical complications included wound infection, bile leakage, pancreatic leakage, anastomotic leakage, etc., while non-surgical complications included bedsores, pneumonia, etc.

Five experts suggested “infection” because it was the most common reason for a long hospital stay and increased expense.

Six experts proposed endpoint indicators such as “hospital stays”, “treatment outcome”, “discharge condition”, and “discharge reason” to reflect the possible risks.

Five experts suggested “arrearage” as an important signal of possible disputes.

Five experts considered the close relationship between hospitalization expenditure and dispute risks.

The indicator system after the first round consultation is summarized in Table A2.

Table A2. Medical risk factors in hospital after first round consultation.

| Category | Factors |
| --- | --- |
| Patient basic information | Age, gender, marriage status, occupation, income, residency, insurance |
| Hospitalization situation | Diagnosis, major/minor hepatectomy, preoperative Child-Pugh classification, the presence of additional surgical procedures, procedure type, times of emergency treatment, and admission in ICU, I, II,III level care, bleeding volume in procedure, surgical complications, non-surgical complications, infection, unplanned reoperation, frequency of hospitalization, hospital stays, hospitalization expenditure, volume of blood transfusion, treatment outcome |
| Discharge situation | Discharge condition, discharge reason, arrearage |

Second round consultation:

Seven experts suggested removing “insurance” because 70% of the patients admitted to the hospital were not Shanghai residents covered by local health insurance.

Five experts noted the interaction between “diagnosis” and “admission in ICU” as well as the difficulty in quantifying indicators. As a result, the two were deleted. “I, II, III level care” was also removed for the same reason.

Three experts suggested excluding “procedure type” because of its connection with the volume of bleeding and blood transfusion.

Five experts proposed removing “hospitalization expenditure” and “treatment outcome” because of the interaction with “discharge situation” and “arrearage.”

The indicator system after the second round consultation for further statistical verification is listed in Table A3.

Table A3. Medical dispute factors of liver cancer surgical treatment.

| Category | Factors |
| --- | --- |
| Patient basic information | Sex, age, marital status, occupation, residency |
| Hospitalization situation | Major/minor hepatectomy, preoperative Child-Pugh classification, the presence of additional surgical procedures, terminal condition*, times of emergency treatment, bleeding volume in procedure, surgical complications, non-surgical complications, infection, unplanned reoperation***,** frequency of hospitalization, hospital stays, volume of blood transfusion |
| Discharge situation | Discharge condition, discharge reason, arrearage |

* Unplanned reoperation: patient receives an unplanned reoperation during the same hospitalization because of direct or indirect complications of the surgical procedure.

Terminal condition refers to the situation in which the physician issues a notice to claim the critical condition of the patient.
